# Cortical Network Response to Acupuncture and the Effect of the Hegu Point: An fNIRS Study

**DOI:** 10.3390/s19020394

**Published:** 2019-01-18

**Authors:** Raul Fernandez Rojas, Mingyu Liao, Julio Romero, Xu Huang, Keng-Liang Ou

**Affiliations:** 1 Human-Centred Technology Research Centre, Faculty of Science and Technology, University of Canberra, Canberra 2617, Australia; julio.romero@canberra.edu.au (J.R.); xu.huang@canberra.edu.au (X.H.); 2 Department of Industrial Engineering and Management, National Kaohsiung University of Science and Technology, Kaohsiung 80778, Taiwan; mygliao@nkust.edu.tw; 3 Department of Dentistry, Taipei Medical University Hospital, Taipei 110, Taiwan; klouyu@gmail.com; 4 Department of Dentistry, Taipei Medical University-Shuang Ho Hospital, New Taipei City 235, Taiwan; 5 School of Dentistry, Health Sciences University of Hokkaido, Hokkaido 061-0293, Japan; 6 Department of Prosthodontics, Faculty of Dentistry, Hasanuddin University, Makassar 90245, Indonesia; 7 Department of Prosthodontics, Faculty of Dentistry, Universitas Gadjah Mada, Yogyakarta 55281, Indonesia; 8 Department of Oral Hygiene Care, Ching Kuo Institute of Management and Health, Keelung 203, Taiwan; 9 3D Global Biotech Inc., New Taipei City 221, Taiwan

**Keywords:** pain, Hegu point, acupoints, connectivity, NIRS, repeated measures, anaesthesia, nociception, pain perception

## Abstract

Acupuncture is a practice of treatment based on influencing specific points on the body by inserting needles. According to traditional Chinese medicine, the aim of acupuncture treatment for pain management is to use specific acupoints to relieve excess, activate qi (or vital energy), and improve blood circulation. In this context, the Hegu point is one of the most widely-used acupoints for this purpose, and it has been linked to having an analgesic effect. However, there exists considerable debate as to its scientific validity. In this pilot study, we aim to identify the functional connectivity related to the three main types of acupuncture manipulations and also identify an analgesic effect based on the hemodynamic response as measured by functional near-infrared spectroscopy (fNIRS). The cortical response of eleven healthy subjects was obtained using fNIRS during an acupuncture procedure. A multiscale analysis based on wavelet transform coherence was employed to assess the functional connectivity of corresponding channel pairs within the left and right somatosensory region. The wavelet analysis was focused on the very-low frequency oscillations (VLFO, 0.01–0.08 Hz) and the low frequency oscillations (LFO, 0.08–0.15 Hz). A mixed model analysis of variance was used to appraise statistical differences in the wavelet domain for the different acupuncture stimuli. The hemodynamic response after the acupuncture manipulations exhibited strong activations and distinctive cortical networks in each stimulus. The results of the statistical analysis showed significant differences (p<0.05) between the tasks in both frequency bands. These results suggest the existence of different stimuli-specific cortical networks in both frequency bands and the anaesthetic effect of the Hegu point as measured by fNIRS.

## 1. Introduction

Pain is a primary neurophysiological function, which enables us to perceive severe discomfort or uncomfortable sensations. The International Association for the Study of Pain defines pain as “an unpleasant sensory and emotional experience associated with actual or potential tissue damage, or described in terms of such damage” [1]. In general, pain serves as a warning mechanism for the brain to act against something wrong in the body and has obvious importance for survival. However, pain is a major concern in society because it is a significant public-health problem and is a burden to the economy [2]. In addition, pain affects our daily activities and decreases our quality of life considerably. Thus, since the early days of our civilization, treatments and remedies have been searched for and proposed to mitigate, reduce, and eradicate pain. One of these methods, which has been developed over the centuries, is acupuncture.

Acupuncture is a practice of treatment based on influencing specific points (acupuncture points or acupoints) on the body by inserting needles [3]. This technique has been mainly used to stimulate and assist the progress of health, improve quality of life, and also used to treat common health problems such as headaches, colds, or fevers. Although the origins of acupuncture remain controversial, it has been accepted as part of traditional Chinese medicine (TCM). The Chinese are known to have practised acupuncture for more than 2000 years [4]. The first written records about acupuncture, the Huang Di Nei Jing, dates from about 200 B.C. [5]. Acupuncture is also known to have physiologic effects related to pain reduction or to induce anaesthesia [6,7,8]. One of the first reported applications of acupuncture anaesthesia was in tooth extractions, and then extended to other types of surgeries such as appendectomy, retinal operation, and knee surgery [4,9].

According to TCM, the aim of acupuncture treatment for pain management is to use specific acupoints to relieve excess, activate qi (or vital energy), and improve blood circulation [10]. In this context, the Hegu point is one of the most used acupoints for this purpose, and it has been linked to having an analgesic effect and used for relieving pain. The Hegu point, also known as “large intestine” 4 (LI4), is a source (Yuan) point located at the midpoint on the radial side of the second metacarpal [11]. In experimental research, LI4 is frequently used as the acupoint for the study of acupuncture anaesthesia [12]. This point is widely used for the treatment of headache, toothache, gastric pain, dizziness, sore throat, and to treat internal organs [4,9,12]. On the assumption that the influence of acupuncture and the analgesic effects of the Hegu point can be both observed through different regulation mechanisms in the cerebral activity, their brain activation and response can be measured by neuroimaging methods.

Functional near infrared spectroscopy (fNIRS) can be used as a non-invasive neuroimaging method to obtain brain activity by measuring real-time changes of oxygenated (oxy-Hb) and deoxygenated haemoglobin (deoxy-Hb). This neuroimaging technique has been used to assess cortical activity in diverse experimental and clinical settings. From breast cancer imaging [13], language and cognitive development in infants [14], pain assessment [15,16], studying the effects of treatments in Alzheimer’s patients [17], to understanding the human brain mechanism of pain perception and nociception [18,19,20]. In addition, fNIRS offers advantages over other technologies (fMRI, EEG, PET), such as it is non-invasive, portable, relatively inexpensive, and provides good temporal and spatial resolution [21,22]. For in-depth reviews with more aspects of fNIRS, the interested reader is referred to [23,24,25].

Although acupuncture has been widely used for pain control and anaesthesia, there exists considerable debate as to its scientific validity [26,27,28]. In this pilot study, we aim to determine the cortical functional connectivity related to the main three types of acupuncture stimulation and identify an analgesic effect based on the hemodynamic response as measured by fNIRS. For that reason, we used acupuncture in eleven (*n* = 11) subjects and measured their cortical hemodynamic response. Wavelet coherence and wavelet transform were used to evaluate the functional connectivity between channel pairs and to explore the anaesthetic effect of acupuncture, respectively. The results showed task-specific brain networks after each stimulus, and the statistical analysis showed a significant difference between the acupuncture stimuli.

## 2. Materials and Methods

### 2.1. NIRS Recording

**Figure 1:**
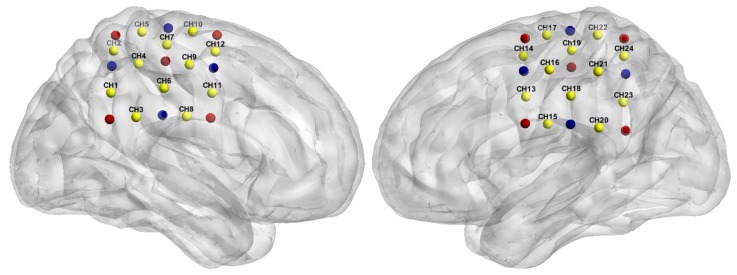
Probe configuration, right hemisphere (Ch1–12) and left hemisphere (Ch13–24); probes were centred on the primary somatosensory region (C3 and C4 positions). Channels (in yellow) are measured between emitters (in red) and receivers (in blue).

Functional NIRS recordings were acquired using a multichannel optical topography system, Hitachi ETG-4000 (Hitachi Medical Corporation, Tokyo, Japan). Since oxy-Hb and deoxy-Hb absorb NIR light differently, this equipment uses two wavelengths of near-infrared light (695 nm and 830 nm) to measure changes of haemoglobin concentration in the cerebral cortex. The system also provides measurements of total haemoglobin (total-Hb) as the sum of oxy-Hb and deoxy-Hb. The spectrometer is equipped with a 24-channel cap, configured as 12 channels per hemisphere (Figure 1). Each hemispheric probe consists of five sources (red circles) and four detectors (blue circles) that provide twelve source-detector pairs. According to the EEG 10–20 system [29], the measuring probes were centred on the C3 and C4 positions [30]. The reason for using this cortical area is that the somatosensory cortex (SI) has been associated with the processing of noxious stimulus input [31,32] and also encoding of information about the location and duration of pain [18]. In this study, we used only the oxy-Hb signals since these signals have a better signal-to-noise ratio than deoxy-Hb signals [33,34]. The sampling rate was 10 Hz.

### 2.2. Stimuli

**Figure 2:**
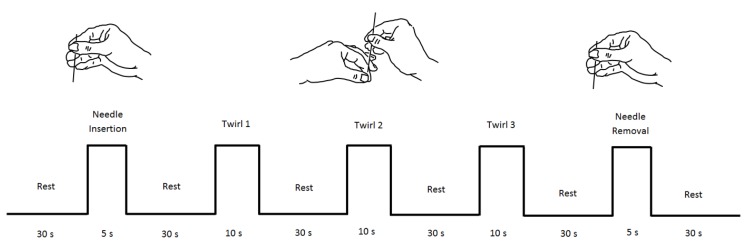
Block sequence of the three different acupuncture manipulations. The first task was needle insertion (NI) followed by an initial twirl to activate the acupoint. The second task was needle twirl, which was repeated three times (T1, T2, and T3) to keep the acupoint activated. The last task was needle removal (NR).

**Figure 3:**
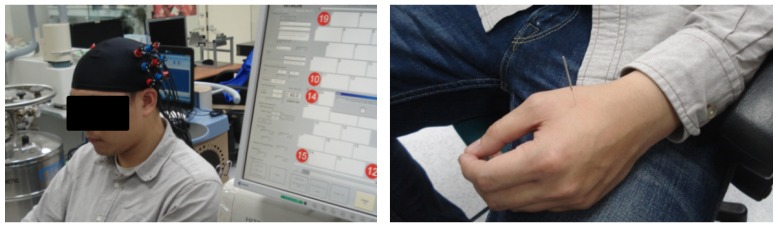
Probe configuration and anatomical location. Subject wearing the multi-channel measuring cap (**left panel**). Acupuncture point (LI-4) used in the study (**right panel**).

Using traditional Chinese acupuncture techniques, all acupuncture manipulations were done by an acupuncturist of Taipei Medical University Hospital. The complete acupuncture procedure is shown in Figure 2, and three different acupuncture techniques (tasks) were used: needle insertion (NI), needle twirl (T1, T2, and T3), and needle removal (NR). The acupuncture point (or acupoint) used in all the experiments was the Hegu point, located on top of the hand, between the thumb and forefinger. One of the advantages of this acupuncture point is its easy access, and the hand can be set aside while the participant is relaxed in a chair. Figure 3 (right panel) presents the anatomical location of stimulation and the participant wearing the fNIRS cap (left panel). All subjects were asked to keep their eyes closed to avoid bias towards the stimulation [35]. Procedures and methods used in this study were conducted in accordance with the guidelines accepted by the Declaration of Helsinki. This research study was approved by the full-board review process of the Taipei Medical University—Joint Institutional Review Board under Contract Number 201307010.

### 2.3. Participants

We considered 11 right-handed volunteers in the study after providing written informed consent. No participants reported a prior history of neurological or psychiatric disorder, a current unstable medical condition, or under medication at the time of testing. The experimental procedure was to the explained participants, and they had the opportunity to stop the procedure at any time if needed. In addition, all the participants expressed that they had never experienced any type of acupuncture treatment, and no incidents were reported during or after the acupuncture procedure. Figure 3 presents the complete experimental arrangement for this study.

### 2.4. Data Analysis

As pre-processing step, to reduce extracerebral hemodynamics (scalp) and systemic variables (e.g., breathing, heart rate, Mayer waves) existed in our data, we followed the method presented by Zhang et al. [36], and we applied principal component analysis (PCA) to the baseline data, assuming that baseline data primarily contain the spatial patterns of systemic interference and identified the first PC (which accounted for approximately 90% of the variance); then, we attenuated the interference in the stimulus data by projecting the stimulus data onto the orthogonal subspace of identified spatial eigenvectors to obtain clean stimulus data. Finally, the time series of each subject was normalized by dividing all of the subject’s channels by the maximum value during the stimulation task. The advantage of using this method is that the intra-variability between channels remains intact while keeping the variability among subjects [37]. In addition, to establish the basis of our framework and introduce the mathematical notations, we present a brief introduction to continuous wavelet transform and wavelet transform coherence; we refer to [38,39] for more detailed information.

#### 2.4.1. Wavelet Transform

Continuous wavelet transform provides a detailed description of the power spectrum of a signal in terms of both the time and frequency domain. Continuous wavelet transforms are useful in signal identification and detection of hidden transients, e.g., hard to detect, short-lived elements of a signal [34]. The classical construction of the wavelet transform requires two main parameters, a time series x(t) and a mother wavelet function ψ(t). Therefore, the continuous wavelet transform of a time-dependent signal x(t) can be defined as:

(1) $$W\left(s,\tau \right)=\frac{1}{\sqrt{s}}\int x\left(t\right)\psi^{*}\left(\frac{t-\tau }{s}\right),$$

where *s* and τ are the varying scale and translation, respectively, the factor $\sqrt{s}$ is for energy normalization across the different scales, and (${}^{*}$) indicates the complex conjugate. Through wavelet transforms, the time series x(t) is projected on the two-dimensional space, scale (*s*), and translation (τ). In our analysis of fNIRS series (or channels), a Morlet wavelet was used as the mother wavelet function, and this function has zero mean and is localized in both the time and frequency space [38]. The Morlet wavelet is defined as:

(2) $$\psi_{0}\left(t\right)=\pi^{-1/4}e^{i\omega_{0}t}e^{-1/2t^{2}}$$

where $\omega_{0}$ is dimensionless frequency, frequently $\omega_{0}\geq 6$ to satisfy the optimal value for the time-frequency resolution [40]. As we chose the Morlet wavelet, $\omega_{0}=6$ is a good choice due to its good balance between time and frequency localization [41]. In particular, wavelet transform was used to obtain the detailed description of the power spectrum of the fNIRS signals and obtain the data for the statistical analysis (refer to Section 2.5).

#### 2.4.2. Wavelet Coherence

Our analysis of functional NIRS signals requires comparing fNIRS signals from opposite cortical regions simultaneously; therefore, wavelet coherence is used to measure how similar two signals are. The coherence value indicates how coherent or correlated two signals are in both the time period and the frequency band. Given two time series, x(t) and y(t) with wavelet coefficients $W_{x}\left(s,\tau \right)$ and $W_{y}\left(s,\tau \right)$, respectively, and the cross-wavelet power given by $W_{xy}\left(s,\tau \right)=W_{x}\left(s,\tau \right)W_{y}^{*}\left(s,\tau \right)$, the wavelet coherence is defined as [41]:

(3) $$WC\left(s,\tau \right)=\frac{{\left|S\left(s^{-1}W_{xy}\left(s,\tau \right)\right)\right|}^{2}}{S\left(s^{-1}{\left|W_{x}\left(s,\tau \right)\right|}^{2}\right)S\left(s^{-1}{\left|W_{y}\left(s,\tau \right)\right|}^{2}\right)},$$

where *S* is a smoothing operator, which is applied by a convolution in time and scale. The resulting values of WC(s,τ) range from 0≤WC(s,τ)≤1. There is a perfect linear relation at a specific time and scale between the two signals when WC(s,τ)=1, and the two signals can be seen as independent when WC(s,τ)=0. The wavelet coherence can be thought as an extension of the Pearson’s correlation; however, the wavelet coherence is a localized correlation coefficient in the time-frequency domain [41].

#### 2.4.3. Bilateral Connectivity Analysis

Bilateral connectivity was studied by comparing the right and left somatosensory cortex during the acupuncture procedure. Using the probe configuration (Figure 2), we compared channels between both hemispheres; for instance, Channel 1 (Ch1) in the left hemisphere with Channel 20 (Ch20) on the right hemisphere, obtaining a total of 12-channel pairs for each comparison. We then applied WTC analysis on each pair of HbOtime series, obtaining a wavelet coherence map for each comparison of the channel pairs. The analysis was divided into two frequency bands, the very-low frequency oscillations (VLFO, 0.01–0.08 Hz) and the low frequency oscillations (LFO, 0.08–0.15 Hz). The reason for using these two frequency bands is that these frequency bands are linked to neural activity related to the period of stimulation [42]. In addition, these frequency bands are less affected by other physiological signals such as respiration (0.16–0.6 Hz) and heartbeat (0.8–1.5 Hz) [43].

### 2.5. Statistical Analysis

In order to assess the anaesthetic effect of the Hegu point, the fNIRS time series were transformed to the time-frequency domain using the wavelet transform as explained in Section 2.4.1. Using the obtained wavelet coefficients in these two bands, the coefficients were averaged from the start of the stimulus up to fifteen seconds after the stimulation. The reason for choosing this window was based on the visual inspection of the hemodynamic response after each stimulus. With these coefficients, a mixed model ANOVA was used to appraise statistical differences in the wavelet domain for the five different stimuli (needle in, Twirls 1–3, and needle out). The null hypothesis behind this analysis was that there were no significant differences between stimuli. A logarithmic transformation was used to both reinforce the linearity of the model (any multiplicative effect is treated as an additive effect instead) and scale the values of the coefficients to a range that avoids numerical problems when running the analysis of variance. Three different methods to assess differences within the groups (pairwise comparisons) were implemented: Tukey, Scheffe, and Bonferroni. As all three methods had similar results, only Tukey’s scores are reported in this paper. Shapiro–Wilk, F${}_{max}$, and Levene’s test statistics were used to test the assumptions of normality and homogeneity of variance. The assumptions for a mixed model ANOVA were not violated. Statistical significance was reported for p<0.05, and the overall effect size was calculated using a partial eta-squared statistic.

## 3. Results

### 3.1. Hemodynamic Response to Acupuncture Stimulation

**Figure 4:**
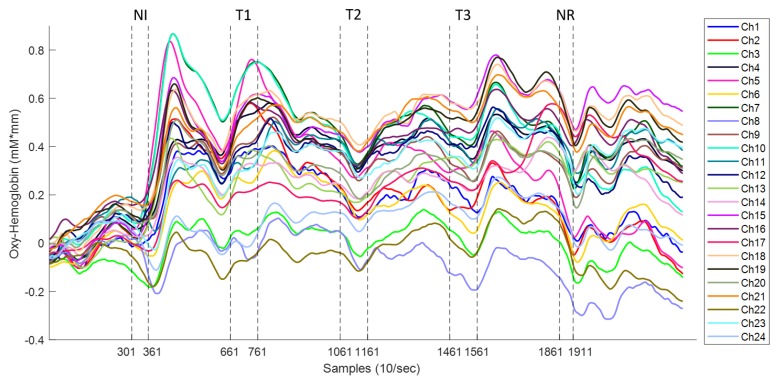
Pain response. Dynamic trace recorded in the 24 channels after each acupuncture stimulation. Vertical dotted lines represent the period of stimulation. The acupuncture manipulations are needle insertion (NI), needle twirls (T1, T2, T3), and needle removal (NR).

Needle insertion (NI) was the first acupuncture manipulation in the experiment. It was applied at thirty seconds after commencement of the experiment and lasted for six seconds. Each acupuncture manipulation was applied within the time indicated by the dotted lines (markers) in Figure 4, and four channels were used only for illustration purposes. Once the insertion was done (within Samples 301 and 361), the needle was twirled until de-qi (Chinese for obtaining the qi or arriving at the qi) was reached. Once the patient manifested numbness/heaviness in the arm, the acupuncturist stopped the stimulation. This de-qi sensation was approximately reached three seconds after needle insertion. Immediately after reaching the highest pain response, approximately after 14 s, the oxy-Hb signal dropped towards baseline. It is worth noting that in all subjects, the highest peak was exhibited after this first stimulus (needle insertion). Needle insertion was followed by a resting time of 30 s. This time was used for the patient to rest after the stimulus and let the hemodynamic response go back to baseline. It is worth mentioning that there was a delay in reaction time between the physical stimulation and the fNIRS signal among all the stimulations, and this is consistent with previous research using fNIRS [44,45,46].

The needle twirls (T1,T2,T3), performed by the acupuncturist, aimed at increasing the de-qi sensation. The time frame for this stimulation was ten seconds and was carried out immediately after resting time; refer to Figure 2. The acupuncturist stopped the twirl manipulation early when the patient expressed full numbness before the ten seconds. Similarly, the signal pain behaviour exhibited a small drop first before increasing constantly for 8 s. At approximately 14 s after the start of the twirl stimulation, a large peak of HbO was obtained. Again, this signal dropped towards baseline after reaching its highest peak. After the stimulus application, the patient was given 30 s to rest, and there was a strong response after the stimulus. It is worth noting that, in Twirl 1 (T1), it appears that there was an early activation before the stimulus; however, this was only seen in channels from the right hemisphere (Ch1–Ch12), while in channels on the left hemisphere (Ch13–Ch24), there was activation after the stimulus; this early activation might reflect the subject’s pain expectation in particular, as it is a contralateral response, since the stimulation was done in the left hand.

The hemodynamics of needle removal (NR) are similar to needle insertion; in this stage, the removal was done within a time frame of five seconds (Samples 1861–1911). This stimulation was carried out immediately after the fourth resting time. In this manipulation, the acupuncturist removed the needle with a fast abrupt pull. Similarly, to all previous stimuli, the pain response to needle manipulation exhibited a small drop of oxy-Hb followed by an increase reaching a peak in approximately four seconds after needle removal. After needle removal, the patient was given 40 s to rest, which was used to let the hemodynamic signals go back to baseline and to end the experiment.

### 3.2. Wavelet Analysis

**Figure 5:**
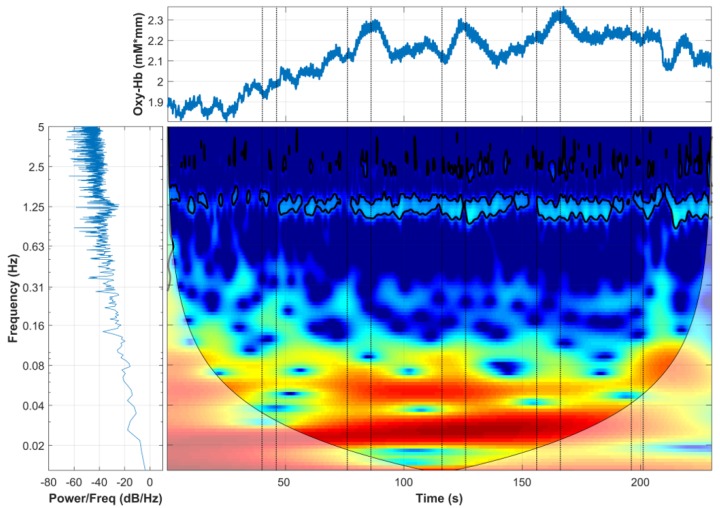
Analysis of a raw oxy-Hb signal using the wavelet transform.The subject’s heartbeat is evident at the frequency of ∼1.25 Hz, and it can be seen as a large peak in the frequency spectrum (**bottom left**), affecting the data during all the experiments, as observed in the wavelet domain (**bottom right**). The effect of a moving artefact after the last stimulus in the time domain is also clear (**top**), which affects several frequency bands (only observed in the wavelet domain).

Wavelet transform was computed for each channel, and the corresponding time-frequency behaviour can be observed. Figure 5 presents a comparison between the domain representations used to generate the wavelet analysis. This image combines the tree domains; the raw oxy-Hb signal in the time domain is presented in the top panel; the power spectrum describing the distribution of power into frequency components composing the oxy-Hb signal is presented in the bottom left panel; and in the bottom right panel, the oxy-Hb signal is exhibited in the wavelet domain, time in the *x* axis and frequency in the *y* axis.

**Figure 6:**
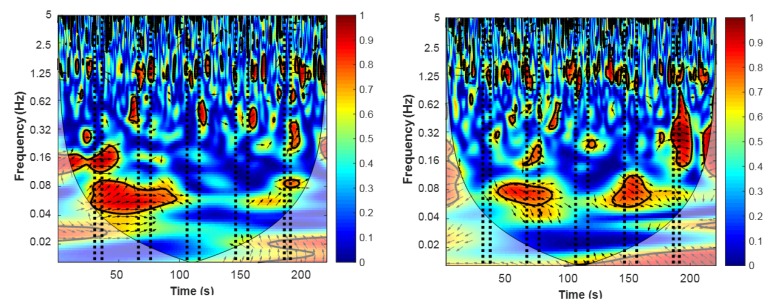
Example of the wavelet coherence transform (WTC) between the raw oxy-Hb signals from Ch3 and Ch20 (**left figure**) and from Ch7 and Ch19 (**right figure**) of a subject. Vertical dotted lines represent the start and finish for each stimulation task. The expectation was that the high coherence between the two raw oxy-Hb signals would represent the synchronization of cortical areas during the experimental stimulation. Red areas represent areas with high correlation, while blue areas represent areas with little correlation. High coherence around the 1.25-Hz band is attributable to the subject’s heartbeat (∼1 Hz).

After computing the wavelet coherence transform from corresponding channel pairs, we obtained a coherence map, and two examples are presented in Figure 6. The coherence (or wavelet correlation) between both raw oxy-Hb signals was higher (red colour) during or immediately after the task blocks. The expectation was that the high coherence between the two raw oxy-Hb signals represents the synchronization of hemodynamic response during the experimental stimulation. This can be seen as two functional areas of the cerebral cortex working similarly (synchronized response) in opposite lobes of the somatosensory region. In addition, the increase in coherence in the VLFO and LFO bands indicated that the coherence increase is task-related. Another band with high coherence is the 1.25-Hz band; however, this high coherence corresponds to the subjects’ heartbeat (∼1 Hz) [34].

### 3.3. Cortical Connectivity

**Figure 7:**
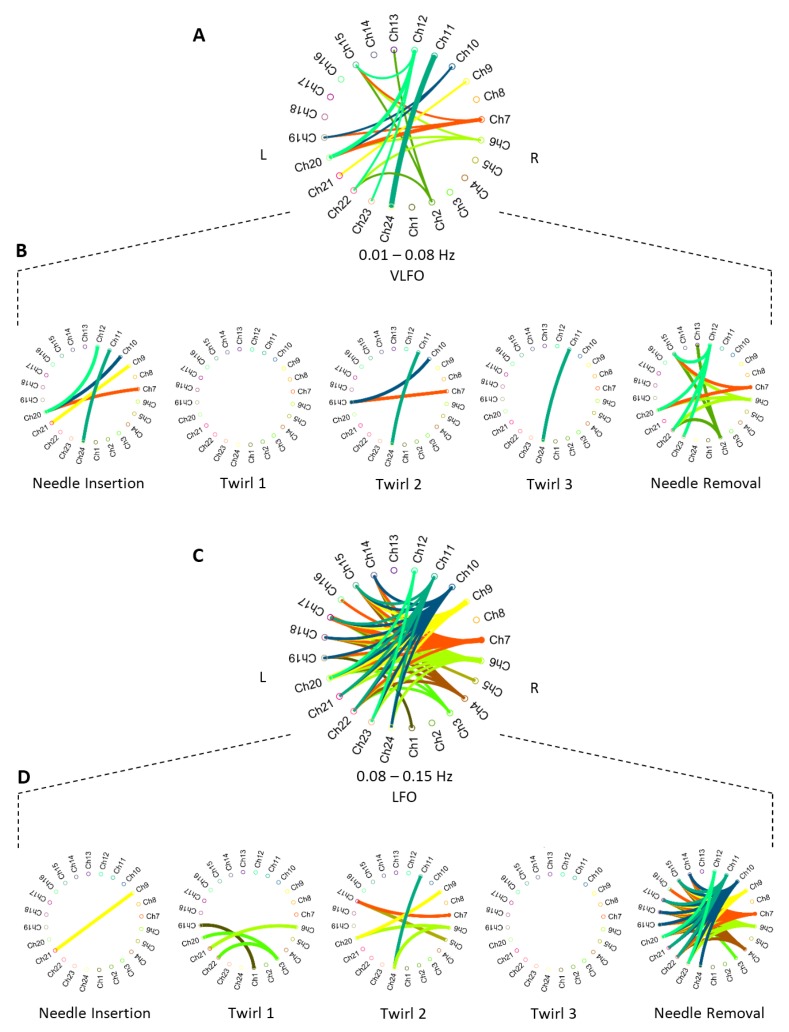
Grand average correlation value for each period band. The top panels (**A**,**C**) show an overall connectivity graph for all the acupuncture tasks in the very-low frequency oscillations (VLFO) and LFO bands, respectively. The bottom panels (**B**,**D**) present the connectivity for each acupuncture manipulation. Each link represents a strong correlation (r>0.7) per each channel pair between the left (L) and right (R) hemispheres.

Based on the activation bands obtained in the wavelet coherence procedure, we can establish the brain connectivity of activated areas. Figure 7 shows the connectivity diagrams between time-frequency correlated cortical areas. The brain connectivity networks were divided into the two distinctive activation bands (VLFO and LFO). In addition, Figure 7B,D shows the cortical interactions of the needle insertion, needle twirls, and needle removal tasks, in each frequency band. It is assumed that the magnitude of a correlation above r>0.7 says more about the strength of the association [47]; therefore, only cortical interactions with strong correlation (r>0.7) were plotted in Figure 7.

We can observe that the channel association in both bands differs in connectivity networks. Firstly, in the VLFO band, Twirl 1 (T1) presented no connectivity among the channels. In the rest of the tasks, strong coherence was observed in two particular pair of channels, the Ch20 and Ch7 interaction in the needle in (NI) and needle removal (NR) tasks; as well as the Ch24 and Ch11 interaction in NI, T2, and Twirl 3 (T3). In addition, Channel 20 appeared to be highly correlated with a number of channels in the opposite hemisphere (Ch7, Ch10, Ch12); the four channels of this cluster are located in close proximity, in the top right section of the topographic map (Figure 1). Secondly, the only task that did not present any interaction was T3 in the LFO band. In the LFO band, there were two main channel interactions: Ch9 was highly correlated with Ch20, Ch21, and Ch23; and Ch20 appeared to be highly correlated with Ch3, Ch9, and Ch12. The interaction between one channel from one hemisphere and a group of channels from the opposite hemisphere during different types of stimuli suggests that these cortical areas operate similarly in response to different stimuli.

### 3.4. Effect of the Hegu Point

**Figure 8:**
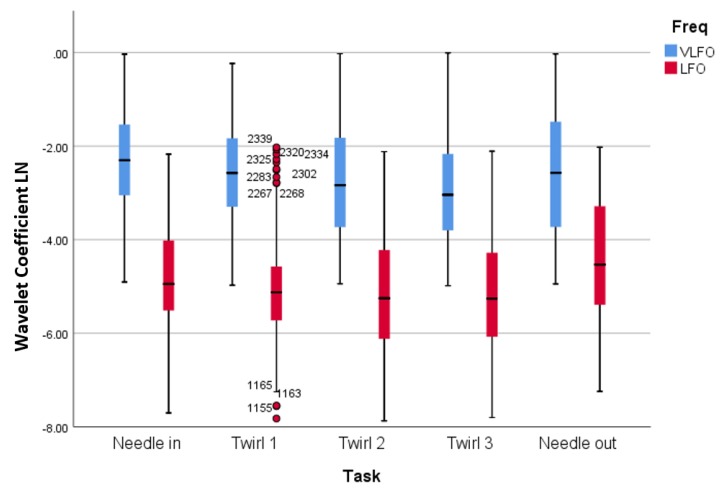
Wavelet distribution according to task and frequency band (VLFO and LFO). Wavelet coefficients are presented in their logarithmic form after transformation. Red dots in Twirl 1 represent outliers in the data.

After computing the wavelet response in each frequency, the wavelet coefficients were averaged within each stimulation time and their respective response time. Figure 8 presents the distribution of the observations according to their responses to the five different stimuli (needle in, Twirl 1, Twirl 2, Twirl 3, and needle out) considering the two frequencies bands (VLFO and LFO). The values are presented in their logarithmic form. In general, the median values of the wavelet coefficients for the VLFO band were higher than the LFO band. In particular, in the VLFO band, the median values presented their highest value during the needle in stimulation and then a steady decrease during the following twirl, to finally have a increment during the needle out stimulus. In the LFO band, the median values presented a similar trend than the VLFO band; however, the highest value was in the last stimulus.

The repeated measures ANOVA analysis indicated significant differences between the acupuncture stimulation. A significant main effect for the VLFO frequency band (refer to Table 1) was measured when applying the different stimuli to the patients F(4,1147) = 7.272, *p*-value < 0.000, partial $\eta^{2}$ = 0.247. Pairwise comparison with needle in was significantly higher than Twirl 2 (MD = 0.439, *p*-value = 0.001; needle in significantly higher than Twirl 3 (MD = 0.535, *p*-value = 0.000); Twirl 1 significantly higher than Twirl 3 (MD = 0.329, *p*-value = 0.025); and Twirl 3 vs. needle out (MD = −0.326, *p*-value = 0.029).

Similarly, a significant main effect was found for the LFO wavelengths (refer to Table 2) recorded F(4,1187) = 15.326, *p*-value < 0.000, partial $\eta^{2}$ = 0.491. The wavelet responses to the needled in task against the Twirl 2 and Twirl 3 stimuli were significantly higher (MD = 0.357, *p*-value = 0.011; MD = 0.353, *p*-value = 0.013), respectively. A significant difference was also found when performing the tasks of needle in and needle out; although, the wavelet response of the former was higher than the latter (MD = −0.381, *p*-value = 0.007). When pulling the needle out, the registered wavelet response was statistically higher than any of the three twirls (MD = −0.659, *p*-value < 0.000; MD = −0.738, *p*-value < 0.000; MD = −0.734, *p*-value < 0.000), respectively.

## 4. Discussions and Conclusions

In this fNIRS study, the stimuli-specific functional connectivity of acupuncture was investigated and the anaesthetic effect of acupuncture in the Hegu point (LI4) was explored. Based on two frequency bands, the very low frequency oscillation (VLFO, 0.01–0.08 HZ) and the low frequency oscillations (LFO, 0.08–0.15 Hz), a wavelet coherence analysis was used to identify functional connectivity, and a repeated measures ANOVA test was used to study the pain response after each manipulation as measured by oxygenated haemoglobin (oxy-Hb). The results showed strong correlations (r>0.7) between different channels and within particular types of stimuli; while the statistical test showed significant differences between stimulus in both bands and an overall decreasing trend after each acupuncture stimulus. These results suggest the existence of different stimuli-specific cortical networks in both frequency bands and the anaesthetic effect of the Hegu point as measured by fNIRS.

The hemodynamic response after the acupuncture manipulations exhibited strong activations. The strongest response was obtained after the needle insertion (NI) and after the needle removal in the VLFO and LFO bands, respectively (Figure 4). However, during the twirls (T1–T3), a decrease of hemodynamic response was observed in both frequency bands. The fact that the hemodynamic response decreased (compared to the first twirl stimulation) can be described by two factors: (1) the subject was familiar with the twirl manipulation and became adapted to this sensation; or (2) by applying pressure in the Hegu point, the subjects experienced an analgesic effect that decreased the effect of pain by the needle manipulations. In addition, the hemodynamic response after the needle removal (NR) increased in both frequency bands; this increment suggests that the pulling of the needle produces a stronger sensation than the needle twirls.

The hemodynamic response was consistent with the results observed by the wavelet analysis (Figure 8), which presented statistical significance between the stimuli (Table 1 and Table 2). The needle insertion presented significantly higher values in the wavelet domain in comparison to Twirl 2 and Twirl 3 in both frequency bands. This observation suggests that the decline of hemodynamic response is also measurable in the wavelet domain. A clear advantage of wavelet analysis is the ability to obtain information from time and frequency simultaneously. For example, in Figure 5 in the time domain (top panel), it is possible to observe a large motion artefact after the 200-s mark, an event that cannot be easily identified without additional analysis in the frequency domain (bottom-left panel). Similarly, a strong physiological signal such as the heartbeat can only be seen in the frequency domain as a large peak in the frequency of ∼1.25 Hz. However, in the wavelet domain (bottom-right panel), these two events can be identified simultaneously without further analysis; hence, the importance of wavelet analysis to study the hemodynamic response in particular frequencies and time points.

Cortical functional connectivity presented distinctive networks after each stimulus in both frequency bands. First, there was no strong connectivity (r>0.7) during Twirl 1 (T1) and Twirl 3 (T3) in either the VLFO or the LFO band. This can be also observed in Figure 8 and the statistical analysis (Table 1 and Table 2); the overall wavelet response of T1 and T3 had a similar response to the previous stimuli (needle insertion and T2) in both frequency bands, and there was no statistical significance in comparison to the previous stimuli in either band. Second, needle removal (NR) presented the largest number of connectivity networks in both frequency bands. In particular, in the LFO band, where most of the connectivity networks were generated, needle removal presented the strongest response in this band (refer to Figure 8). Third, distinctive channel associations were observed between different groups of channels; for instance, in the VLFO band, the strong correlation between Ch20 and a cluster of channels (Ch7, Ch10, Ch12) on the opposite hemisphere. Similarly, in the LFO band, a connectivity network was found between Ch9 and a cluster of channels (Ch20, Ch21, Ch23). These two connectivity networks were observed during the needle insertion (NI) and NR tasks, which suggests a relationship between the size of the connectivity networks and the intensity of the stimulus, since in the wavelet domain (Figure 8), these two tasks presented the highest response in both frequency bands. In addition, the cluster formed in the right hemisphere (Ch7, Ch10, Ch12) is located near the motor cortex, while the cluster in the left hemisphere (Ch20, Ch21, Ch23) is located on the sensory cortex; the connectivity between channels in these two distinctive areas suggests that during the stimulations, there exists an involuntary response against pain or the reflex arc [48]. Finally, these strong relationships between different clusters of channels suggest that two cortical areas in opposite hemispheres (bilateral connectivity) work in a similar or complementary fashion. Therefore, the connectivity networks between these two dominant channels (Ch20 and Ch9) could tell more about the brain functioning as a system during the acupuncture procedure, rather than cortical areas working independently [49].

The use of acupuncture as a treatment of pain has been the subject of debate for decades. Research has shown that acupuncture treatments involve the stimulation of the nervous system [50]. In this study, by measuring the hemodynamic response in the cerebral cortex, the results showed a statistically-significant decline of cortical activity after each acupuncture manipulation (refer to Figure 8). Although this decrease can be linked to pain adaptation by the subjects, this decrease could also suggest an analgesic effect due to acupuncture. In particular, because each acupuncture procedure (apart from needle out) aimed at de-qi (or increase the qi) in the subjects, when de-qi was achieved, all the participants reported a numb sensation, and this numbness is associated with the anaesthetic effect of acupuncture in traditional Chinese medicine [51,52,53]. Based on this assumption, we hypothesize that the decrease in hemodynamic response is due to the anaesthetic effect of acupuncture in the Hegu point. These results are in line with previous studies: Stacher et al. [54] showed that acupuncture increased the pain threshold in twelve subjects. In another study, Ishimura et al. [55] demonstrated the analgesic effect of acupuncture in shallow tissue by measuring pain thresholds of skin and fascia. In a study of lower back pain, acupuncture exhibited better analgesic effects for symptom improvement than medication [56]. These studies also reflect the idea that changes in sensory thresholds are affected by the acupuncture mechanisms of the nervous system [57].

This study presents both insights into the hemodynamic response to acupuncture and tries to identify the anaesthetic effect of the Hegu point, however, it also presents some limitations. A potential issue was the lack of control for any skin blood flow contributions and intracerebral hemodynamics to the fNIRS signals. Recent studies have highlighted the issue that fNIRS signals encompass not only hemodynamic fluctuations due to neurovascular coupling, but also due to skin blood flow and task-related systemic activity of cortex [58]. In order to mitigate this spurious effect, a PCA denoising method has been applied as pre-processing step [36]. However, this method might not be enough to reduce such sources of hemodynamic interference, and other methods, such as short-separation channels or the use of additional instruments to measure physiological information, might be needed [15]. Another limitation is the fact that the acupuncturist might influence the pain perception and the somatosensory processing. Research has shown that the neural processing of pain and experience of pain are affected by expectations and attention [59]. Although, the subjects kept their eyes closed before each stimulus, to reduce visual stimulation and prevent pain expectation, the acupuncturist’s manoeuvres were present; in particular, because the subjects could feel the needle-guide tube before the needle is tapped over the skin. Therefore, this could potentially influence the stimulation effect and bias the cortical response due both to the subject’s expectation of pain before the stimuli and to the contributions of brain activity relating to sensory processing in the somatosensory cortex [35]. A possible approach to overcome this limitation might be the use of control conditions such as using cotton swaps to account for the sensory bias produced by the acupuncturist.

Finally, future research directions could improve the validity of this study; for example, the use of more channels to investigate different cortical areas and obtain bigger networks; in addition, the use of different acupuncture points to compare the results between them and their effect in mitigating pain. Furthermore, future research will explore the effect of the Hegu point in diminishing pain against real medical conditions, such as acute and chronic pain, neuropathic pain, or fibromyalgia.

## Figures and Tables

**Table 1:** Pairwise comparison VLFO, *p*-value numbers marked in bold indicate numbers that are significant on the 95% confidence limit.

Task		Mean Difference	Standard Error	*p*-Value	95% Confidence Interval	
					Lower Bound	Upper Bound
Needle in	Twirl 1	0.206	0.112	0.354	−1.101	0.512
	Twirl 2	0.439	0.110	**0.001**	0.137	0.740
	Twirl 3	0.535	0.111	**0.000**	0.231	0.839
	Needle out	0.208	0.113	0.347	−0.100	0.517
Twirl 1	Twirl 2	0.233	0.111	0.214	−0.068	0.534
	Twirl 3	0.329	0.111	**0.025**	0.026	0.632
	Needle out	0.003	0.112	1.000	−0.305	0.310
Twirl 2	Twirl 3	0.096	0.109	0.904	−0.202	0.395
	Needle out	−0.230	0.111	0.231	−0.533	0.073
Twirl 3	Needle out	−0.326	0.112	**0.029**	−0.631	−0.021

**Table 2:** Pairwise comparison LFO, *p*-value numbers marked in bold indicate numbers that are significant on the 95% confidence limit.

Task		Mean Difference	Standard Error	*p*-Value	95% Confidence Interval	
					Lower Bound	Upper Bound
Needle in	Twirl 1	0.278	0.110	0.086	−0.023	0.580
	Twirl 2	0.357	0.000	**0.011**	0.055	0.659
	Twirl 3	0.353	0.111	**0.013**	0.050	0.656
	Needle out	−0.381	0.113	**0.007**	−0.689	−0.073
Twirl 1	Twirl 2	0.079	0.111	0.955	−0.225	0.383
	Twirl 3	0.074	0.112	0.964	−0.231	0.380
	Needle out	−0.659	0.114	**0.000**	−0.970	−0.349
Twirl 2	Twirl 3	−0.004	0.112	1.000	−0.310	0.301
	Needle out	−0.738	0.114	**0.000**	−1.049	−0.427
Twirl 3	Needle out	−0.734	0.114	**0.000**	−1.046	−0.422
